# Modelling the Interplay between Lifestyle Factors and Genetic Predisposition on Markers of Type 2 Diabetes Mellitus Risk

**DOI:** 10.1371/journal.pone.0131681

**Published:** 2015-07-08

**Authors:** Celia G. Walker, Ivonne Solis-Trapala, Christina Holzapfel, Gina L. Ambrosini, Nicholas R. Fuller, Ruth J. F. Loos, Hans Hauner, Ian D. Caterson, Susan A. Jebb

**Affiliations:** 1 MRC Human Nutrition Research, Elsie Widdowson Laboratory, Cambridge, United Kingdom; 2 Else Kroener-Fresenius-Center for Nutritional Medicine, Faculty of Medicine, Technische Universität München, Munich, Germany; 3 Boden Institute of Obesity, Nutrition, Exercise and Eating Disorders, University of Sydney, Sydney, NSW, Australia; 4 The Charles Bronfman Institute for Personalized Medicine, The Mindich Child Health and Development Institute, The Genetics of Obesity and Related Metabolic Traits Programme, The Icahn School of Medicine at Mount Sinai, New York, New York, United States of America; University College Dublin, IRELAND

## Abstract

The risk of developing type 2 diabetes mellitus (T2DM) is determined by a complex interplay involving lifestyle factors and genetic predisposition. Despite this, many studies do not consider the relative contributions of this complex array of factors to identify relationships which are important in progression or prevention of complex diseases. We aimed to describe the integrated effect of a number of lifestyle changes (weight, diet and physical activity) in the context of genetic susceptibility, on changes in glycaemic traits in overweight or obese participants following 12-months of a weight management programme. A sample of 353 participants from a behavioural weight management intervention were included in this study. A graphical Markov model was used to describe the impact of the intervention, by dividing the effects into various pathways comprising changes in proportion of dietary saturated fat, physical activity and weight loss, and a genetic predisposition score (T2DM-GPS), on changes in insulin sensitivity (HOMA-IR), insulin secretion (HOMA-B) and short and long term glycaemia (glucose and HbA1c). We demonstrated the use of graphical Markov modelling to identify the importance and interrelationships of a number of possible variables changed as a result of a lifestyle intervention, whilst considering fixed factors such as genetic predisposition, on changes in traits. Paths which led to weight loss and change in dietary saturated fat were important factors in the change of all glycaemic traits, whereas the T2DM-GPS only made a significant direct contribution to changes in HOMA-IR and plasma glucose after considering the effects of lifestyle factors. This analysis shows that modifiable factors relating to body weight, diet, and physical activity are more likely to impact on glycaemic traits than genetic predisposition during a behavioural intervention.

## Introduction

Type 2 diabetes mellitus (T2DM) develops as a consequence of the interplay between genetic and lifestyle factors. There is known to be a strong heritable component for T2DM with genetic factors explaining around 25% of the variation in disease risk and approximately 60% of variation in impaired glucose tolerance [1]. Genome-wide association studies (GWAS) have identified many single nucleotide polymorphisms (SNPs) consistently associated with an increased risk for T2DM [2–6] with many of these and additional SNPs associated with insulin secretion and glycaemic traits [7, 8]. Overweight and obesity, physical inactivity, and diets with a high proportion of saturated fat and low non-starch polysaccharide (NSP) are the lifestyle factors identified with convincing or probable evidence of increased risk of developing T2DM [9]. Conversely, weight loss has been shown to improve insulin sensitivity and glycaemic control in people with impaired glucose tolerance or T2DM [10, 11], and when combined with reductions in saturated fat, increases in dietary fibre and increases in physical activity can reduce the incidence of developing T2DM [12–14]. However, there is substantial inter-individual variation in the improvements in insulin sensitivity and glycaemia for a given change in lifestyle factors which may reflect intrinsic characteristics such as genotype.

When analysing the effectiveness of lifestyle changes in lowering disease risk it is difficult to represent and take into account this multitude of factors in a single statistical model. Conventional analysis usually focusses on *individual response variables*, separately, and their relationship to other factors (for example diet and exercise, or physical activity and genetic predisposition), whilst considering other factors as confounding variables. However, a model allowing for *multiple response variables* would be more suited to assess relative associations reflecting the interrelationships amongst all the variables. The aim of this study is to describe the complex effect of changes in lifestyle factors known to impact on the risk of developing T2DM (weight, diet and physical activity) whilst considering genetic predisposition and other intrinsic factors on changes in glycaemic traits in overweight or obese participants following 12-months of a weight management programme. To achieve this we have taken a novel approach using a graphical Markov model to explore the paths of association between changes in weight, physical activity, proportion of dietary saturated fat and NSP in response to a 12 month weight loss intervention, as well as intrinsic characteristics such as age, sex and genetic predisposition to T2DM on the change in glycaemic traits. The lifestyle factors were chosen as those for which there is convincing or probable evidence of an increased risk for developing T2DM by the World Health Organisation [9]. The intrinsic factors chosen were SNPs which have been identified in GWAS associated with an increased risk of T2DM [2–5, 7, 15, 16] along with age and sex. Graphical Markov modelling has proved to be an effective tool to investigate paths of associations in studies which include many variables from each individual [17, 18] and are particularly valuable at depicting hypothetical associations, estimating these associations, and conveying the mechanistic link. For example this method has been applied to evaluate complex relationships between clinical, social and economic variables affecting outcomes of mental health care [19].

## Materials and Methods

### Original trial study design

Full details of the original intervention trial (ISRCTN: 85485463) have been published previously [11]. Briefly, this trial was conducted in 3 countries: Australia, Germany and the UK and compared 12 months of free access to a commercial weight loss programme (CP) to standard treatment in primary care (SC) as defined by national treatment guidelines for each country. Adult overweight and obese participants (n = 772, BMI 27–35 kg/m^2^) who also had at least one risk factor for obesity-related disorders were randomised into the trial. Ethical approval for the study was granted by the Ethics Review Committee of the Sydney South West Area Health Service, NSW, Australia, the Ethical Committee of the Faculty of Medicine of the Technische Universität München, Germany and Nottingham Research Ethics Service, UK. Written informed consent from participants was obtained including for the subsequent genetic analyses.

### Study cohort

In total 353 participants were included in the current analysis. Of the 772 participants who enrolled in the study 442 (57%) were measured at 12 months. To reduce heterogeneity in genetic background only individuals of white European ancestry based on self-reported ethnicity were included in this analysis (n = 402; 91%). DNA was available and met the quality control criteria for 370 of these participants (n = 32 excluded). For the purposes of this analysis, participants on medication for T2DM (n = 17) were excluded.

### Participant measurements

Body weight was measured at baseline and at the end of the 12 month study. Fasting blood was also taken at baseline and 12 months to measure HbA1c, plasma glucose and insulin. In Australia, analyses were conducted at Laverty Pathology (Sydney, New South Wales, Australia). Glucose was measured by a hexokinase method (Siemens Advia 2400, Siemens Australia, Bayswater, Australia); insulin with a chemiluminescent immunoassay (Siemens Advia Centaur, Siemens Advia 2400, Siemens Australia, Bayswater, Australia); HbA1c was measured by HPLC (Bio-Rad turbo-II, CA, USA). In Germany, all analyses were carried out at Synlab Medizinisches Versorgungszentrum Labor München Zentrum GbR (Munich, Germany). Glucose was measured by a hexokinase method (Modular DPE, Roche Diagnostics GmbH, Mannheim, Germany); insulin with an electrochemiluminescence immunoassay method (Immulite 2000, Siemens Healthcare Diagnostics GmbH, Eschborn, Germany); and HbA1c with the Tina-quant turbidimetric immunoassay (Integra 800, Roche Diagnostics GmbH, Mannheim, Germany). In the UK, insulin analyses were conducted by the NIHR Cambridge Biomedical Research Centre, Core Biochemical Assay Laboratory, Addenbrookes Hospital (Cambridge, UK) using the 1235 AutoDELFIA automatic immunoassay system using a two-step time resolved fluorometric assay. All other UK analyses were conducted at Northampton General Hospital (Northampton, UK). Glucose was measured by a colorimetric assay using a glucose oxidase method and HbA1c was measured by HPLC (Bio-Rad turbo-II, CA, USA). HOMA-B and HOMA-IR were calculated for all individuals using the HOMA2 calculator (http://www.dtu.ox.ac.uk/homacalculator/index.php).

### SNP selection and genotyping

All participants were genotyped using a panel of 25 SNPs within 24 loci. The genotyped SNPs had been shown in previous GWAS to be associated with T2DM or fasting plasma glucose or were proxies for the lead SNPs (LD r^2^>0.9, S1 Table). Of these SNPs 11 have previously been associated with glycaemic traits [7, 8, 20, 21] as detailed in **S1 Table**. Samples were genotyped with the Mass ARRAY system using the iPLEX Gold Chemistry (Sequenom, San Diego, CA, USA). The samples were analysed in a matrix-assisted laser desorption ionization time of flight mass spectrometer (MALDI TOF MS, Bruker Daltonik, Leipzig, Germany). The minor allele frequency in our sample was consistent with previous studies [2–5, 7, 15, 16]. SNPs with a call rate of <95% were excluded from analyses (4 SNPs: MTNR1B, rs10830963; NOTCH2, rs10923931; IGF2BP2, rs1306077; WFS1, rs10010131). Individuals were excluded if genotyping was unsuccessful in ≥3 SNPs (n = 32). No SNPs were excluded for deviation from the Hardy-Weinberg Equilibrium using the log likelihood ratio chi-square test (1 df) for association using a cut-off of P = 0.002, based on a Bonferroni correction for 25 tests. Two SNPs within the CDKN2B locus were included in this analysis as they were not in linkage disequilibrium (r^2^ = 0.0). As such 21 SNPs in 20 loci were included in the analyses of the 353 remaining participants (**S1 Table**).

### T2DM genetic predisposition score (T2DM-GPS)

We defined genetic predisposition using a score generated from individual SNPs associated with T2DM in GWAS. We defined the risk-allele as that associated with T2DM incidence or higher fasting plasma glucose levels in previous GWAS [2–5, 7, 15, 16]. An individual’s genotype was coded as 0, 1 or 2 depending on the number of risk alleles for that SNP and a genetic predisposition score (T2DM-GPS) was calculated by adding the number of risk alleles (**S1 Table**). We adopted a simple addition of the associated risk alleles for each trait following others [22–24] because it shows consistent results with weighted risk scores [25, 26], whilst allowing a more straightforward interpretation. For participants missing individual genotyping data, the average count of risk alleles for the respective SNP was substituted for the purposes of calculating the T2DM-GPS. No participants had missing data for more than 3 genotypes. The T2DM-GPS ranged from 15–29 with a median value of 22.

### Dietary assessment

Participants were asked to complete a 4-day food diary, using household measures for portion sizes, at baseline and at 12 months. In Australia, completed food diaries were coded using Foodworks 2007 (Xyrius Software. FoodWorks Professional. Brisbane, Australia) and average daily intakes of energy, macronutrients, saturated fat and dietary fibre by the AOAC method were estimated using Australian food composition tables (NUTTAB 2006) [27]. An equivalent NSP value was estimated from the AOAC dietary fibre values using the conversion factor of 0.75. In Germany, food diaries were coded using the commercially available software programme Prodi 5.4.0.0 expert (Nutri-Science GmbH, Freiburg, Germany) based on the German Nutrient Database (BLS) to estimate average daily nutrient intakes of energy, macronutrients and saturated fat, but no measure of dietary fibre was available. In the UK, completed food diaries were coded and linked to British food composition data to estimate average daily intakes of energy, macronutrients, saturated fat and NSP using the DINO (Diet In, Nutrients Out) in-house programme at MRC Human Nutrition Research, Cambridge [28].

Changes in percent of energy from saturated fat between baseline and 12 months were calculated from these average values for each centre.

### Physical activity assessment

To obtain an indication of the change in physical activity, participants were provided with a pedometer (Weight Watchers International, Inc, New York, NY, USA) and instructed to wear it for the duration of the day over a 7-day period at baseline and the end of the study as described previously [29]. The number of steps per day were recorded in the participants’ activity diary and the activity over the 7 days was averaged. The change in activity over the twelve months was calculated as the difference in average number of steps (x1000) between baseline and 12 months.

### Statistical analysis

**Fig 1:**
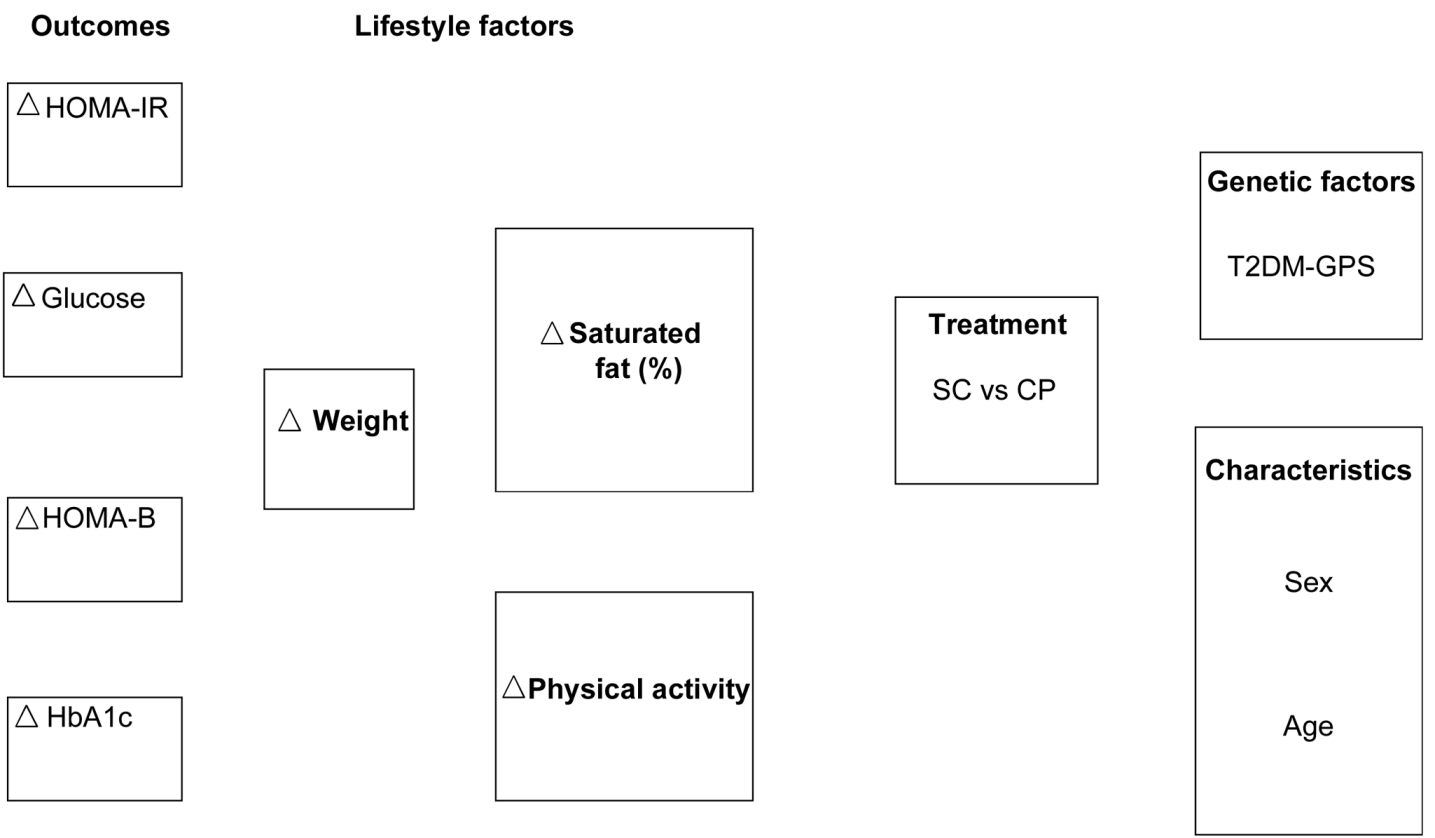
Variables to explore the paths between lifestyle and genetic factors on change in outcomes. Initial ordering of variables including baseline characteristics, genetic factors, treatment, change in lifestyle factors (weight, saturated fat and physical activity) and the change in outcomes. The variables depicted in the figure are ordered so that those variables on the left hand side can be regarded as response variables to those on the right hand side. Some variables play a similar role in the path of associations but they are not related with each other. These are presented in stacked boxes (e.g. dietary saturated fat and physical activity).

Changes in measures of HOMA-IR, HOMA-B, glucose and HbA1c after 12 months were defined as the main outcomes. After preliminary screenings of the sampling distribution of these variables, a logarithmic transformation was adopted to obtain more symmetric distributions of the data. In the logarithmic scale, change at 12 months from baseline is equivalent to the ratio of the 12-month measurement and the baseline measurement. An increase in the levels of an outcome measurement at 12 months would be reflected by a ratio greater than 1 (or 0 in the log scale). Although the outcome variables are interrelated, we analysed them separately using graphical Markov modelling, which extend path analysis, to elucidate the mechanisms that drive changes in each independently [30, 31]. For a given outcome, the primary aim was to trace the effects of treatment group on dietary intake and physical activity which might lead to changes in weight and subsequent changes in outcome in relation to the effects of genetic predisposition and other intrinsic characteristics (Fig 1). The complexity of the model was reduced by ordering the variables according to the order of exposure with characteristics which are intrinsic to the individual (age, sex and genetic predisposition) at the right hand side through to the change in outcomes on the left. In this way the model focused on the type of associations that are clinically relevant, with variables on the left taken as response variables to those located on their right-hand side. To build the graphical Markov model a linear least-square regression model was fitted for each response component of the initial arrangement of variables (Fig 1) on all the variables on its right hand side. The regression model that best described the data was selected by comparing nested models with different combinations of explanatory variables. Graphical Markov models are characterised by a graph. To build the graph, an arrow was added from each of the selected explanatory variables to the response to represent a directed association. For the pair of variables in the last box including personal characteristics, a solid line was added if there was an association between them. In the final graph, arrows indicate the direction of the association, with explanatory variables pointing at their dependent variable directly, or through intermediate explanatory variables connected by arrows, whilst lines with no arrow represent symmetric associations. Any two variables which are not connected correspond to an independence statement. In building the model, we assessed and interpreted interactions that were of clinical relevance (for example testing for an interaction between T2DM-GPS and weight loss is interesting in the context of an improvement in T2DM risk, whereas an association between sex and T2DM-GPS is not). All variables used in analyses were continuous apart from sex and country which were categorical.

We note that the model fitting involved use of multiple statistical tests; however, the components of the model were carefully chosen to reflect distinctive relationships whose interpretation were of interest on their own. In this case the interpretation of each significance level reported is valid and adjustment for multiple testing is not required [32]. Data were analysed using the statistical software R (http://www.r-project.org/).

## Results

### Participant characteristics

The characteristics of the 353 participants who were included in this current analysis, at baseline, and following 12 months on the intervention are given in **Table 1**. There were no differences at baseline between the SC and the CP treatment arms for any of the characteristics and no differences in the distribution of participants taking medications such as beta-blockers, ARBs and calcium channel inhibitors and statins. As reported for the full cohort in the primary analysis [11], the mean change in weight, glucose, HOMA-B and HOMA-IR in response to the intervention were all significantly greater in the CP compared to the SC treatment groups (**Table 1**) in this subset of participants.

**Table 1:** Characteristics of participants who were included in this analysis at baseline and the change after 12 months intervention.

	SC	CP
***n***	174	179
**Age (years)**	50.1 (12.1)	48.5 (13.1)
**Female (%)**	85.0	88.5
**Impaired glucose regulation (%)^a^**	15.1	17.5
**Baseline BMI (kg/m^2^)**	31.3 (2.6)	31.4 (2.6)
**Baseline glucose (mM)**	4.98 (0.57)	4.97 (0.63)
**Baseline insulin (pM)^b^**	47.9 (35.5)	48.0 (32.7)
**Baseline HbA1c (mmol/mol)**	37.8 (4.1)	37.8 (4.1)
**Baseline HOMA-B (%)**	98.8 (33.5)	99.2 (36.6)
**Baseline HOMA-IR^b^**	0.87 (0.66)	0.88 (0.61)
**Δ Weight (kg)**	-3.49 (4.88)	-7.10 (6.56)^c^
**Δ Glucose (mM)**	0.02 (0.49)	-0.11 (0.47)^c^
**Δ Insulin (pM)**	0.48 (26.4)	-7.78 (29.4)
**Δ HbA1c (mmol/mol)**	-0.16 (0.03)	-0.19 (0.03)
**Δ HOMA-B (%)**	-0.33 (3.5.5)	-8.14 (32.3)^c^
**Δ HOMA-IR**	0.01 (0.49)	-0.14 (0.52)^c^

a. Glucose>6.1 mM.

b. data presented as median (SD). All other data presented as mean (SD).

c. Significant differences between standard care (SC) and commercial programme (CP) groups (P<0.05).

### Confirmation of the association between T2DM-GPS and baseline traits

The effect of the T2DM-GPS was tested on the traits at baseline in this cohort to confirm the suitability of the T2DM-GPS as an indicator of T2DM risk in this small cohort of participants. T2DM-GPS was associated with higher fasting plasma glucose (0.04 mM Standard error (SE) = 0.01 mM per risk allele, P<0.0001) and HbA1c (0.21 (SE = 0.07) mmol/mol per risk allele, P = 0.003) and lower insulin secretion (HOMA-B: -1.30 (SE = 0.65) % per risk allele, P = 0.05) (**S1 Fig**). There was no association of T2DM-GPS with insulin resistance (HOMA-IR: -0.00 (SE = 0.01) units higher per risk allele, P = 0.87) (**S1 Fig**). This T2DM-GPS explained 16.8% of the variance in baseline glucose, 3.01% of HOMA-IR, 7.02% of HOMA-B and 25.9% of HbA1c when adjusted for age, sex, country and BMI.

### Dietary factors incorporated in the model

Data for NSP was not available for all participants since it is not a core component of the dietary analysis programme used in Germany. However, based on the data from participants in the other two countries (n = 196) no associations were found between NSP and any of the outcomes indicating that NSP may not be an important factor on the change in outcomes in this study. Therefore we omitted NSP from the analyses and concentrated on the change in proportion of saturated fat as the key dietary factor hypothesised to have a relationship to glycaemic traits.

### Impact of lifestyle factors and genetic predisposition to type 2 diabetes on change in traits

**Fig 2:**
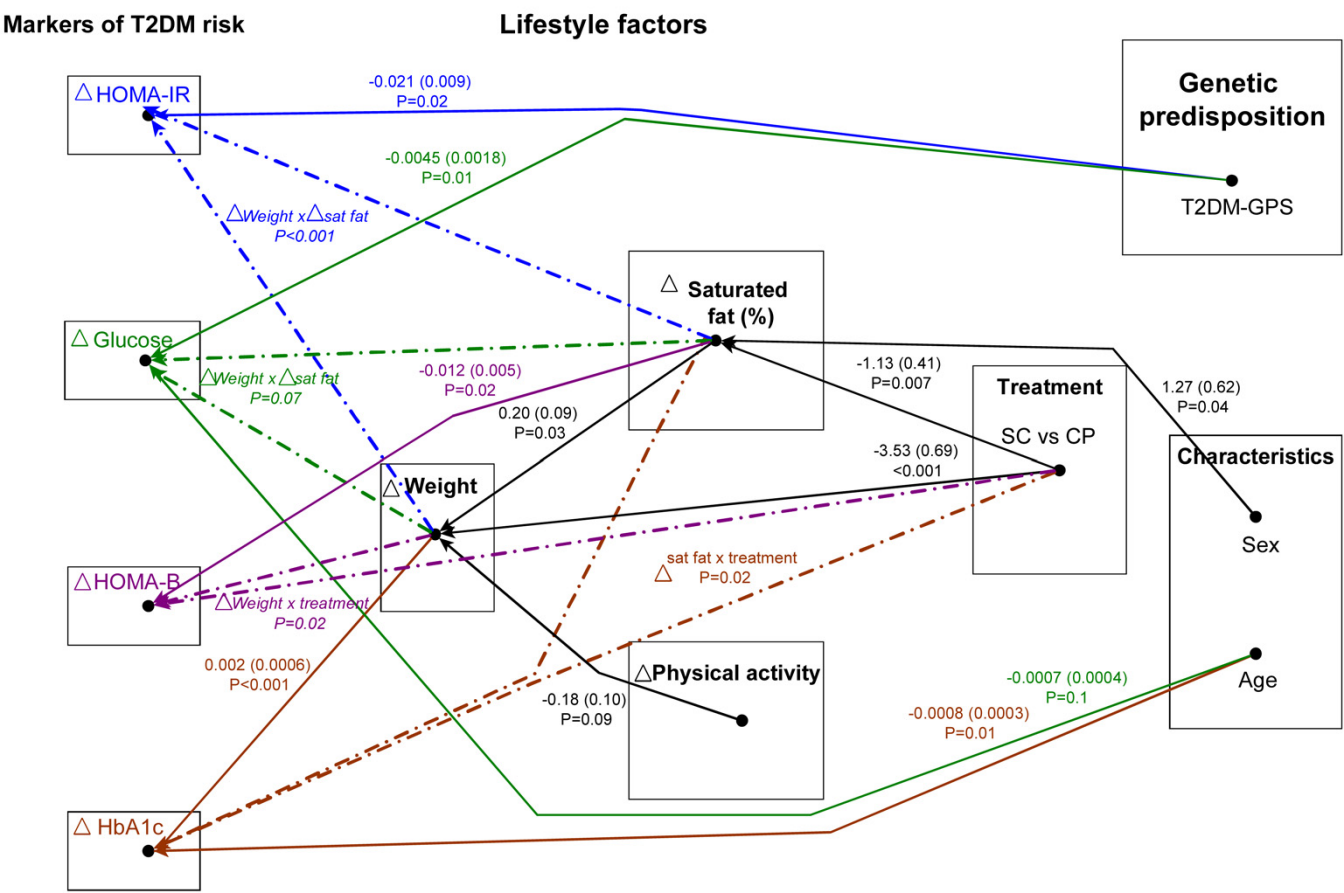
Final graphical Markov models estimated using sequences of regressions. Paths of associations were estimated for changes at 12 months from baseline in HOMA-IR (shown in blue), plasma glucose (shown in green), HOMA-B (shown in purple) and plasma HbA1c (shown in brown). Paths common to all outcomes are shown in black. The arrows represent strong or moderate associations at a significance level of 10%. The strengths of these associations are shown as partial regression coefficients (SE) and P-value for each pair of variables. The partial regression coefficients correspond to changes in the ratio (final/baseline) of the outcome variables in the logarithmic scale. Absence of a line between two variables indicates that they are not associated after conditioning out their combined set of explanatory variables. Significant interactions which were identified are also indicated with dot-dash lines and are detailed in Fig 3. Differences by countries were found, but the effects are not presented for clarity of main findings in the figure. The German centre had significant differences in mean measurements compared to the UK centre for the change in proportion of dietary saturated fat (P<0.1) and physical activity (P<0.1). There were also important differences between the Australian and UK centres in changes of plasma glucose (P = 0.001). Δ Weight (kg), Δ dietary saturated fat (% of total energy), Δ physical activity (1000 000 steps), genetic predisposition (T2DM-GPS, per increased risk allele), age (years), Δ glucose (mM) and Δ HbA1c (mmol/mol).

The final graphical Markov models of dependencies are shown in Fig 2, with different colours to highlight the paths important for the change in each trait, paths common to all traits are shown in black and the presence of interactive terms is indicated by dash-dot lines. The relative strength of the associations was measured by the coefficients of the linear regressions (Fig 2). Their estimated values are shown in the figure if their level of significance is below 0.10 to capture all the relevant information. In this section we describe how to read along the paths of association back from the change in each outcome to the factors which influence that change, directly or indirectly. Some paths are common to each trait and are described in a separate section to avoid repetition. Any interactions identified are described in the following section.

#### HOMA-IR

The paths important for changes in HOMA-IR are shown in blue and black in Fig 2. The factors identified were T2DM-GPS, change in weight and change in saturated fat and their associated common paths. There was also a significant interaction between weight loss and the change in proportion of dietary saturated fat. This model explained 17.8% of the variance in the change in HOMA-IR. To depict the effect of genetic susceptibility we used a fixed mean change in proportion of saturated fat and weight and input low and high values for T2DM-GPS. The model shows that participants with a low T2DM-GPS of 20 (lower quartile) had a reduction in HOMA-IR of 7% whereas participants with a high T2DM-GPS of 24 (upper quartile) had a reduction in HOMA-IR of 15%.

#### HOMA-B

The paths important for changes in HOMA-B are shown in purple and black in Fig 2. An interaction between weight loss and treatment group, as well as the change in the proportion of dietary saturated fat and its common path were identified. This model was found to account for 6.84% of the variance of change in HOMA-B.

#### Plasma glucose

The paths important for changes in plasma glucose are shown in green and black in Fig 2. Weight loss, change in proportion of dietary saturated fat (and their associated common paths), T2DM-GPS, age and country were identified. There was also an interaction between weight loss and the change in proportion of saturated fat in the diet. This model explained 15.7% of the variance of change in glucose. To depict the effect of genetic susceptibility we used a fixed mean change in proportion of saturated fat, weight and average age of participants. The model shows that for participants with a low T2DM-GPS of 20 (lower quartile) there was no change (0.05%) in glucose while for participants with a high T2DM-GPS of 24 (upper quartile) there was a reduction in glucose of 1.84%.

#### HbA1c

The paths important for changes in HbA1c are shown in brown and black in Fig 2. In addition to weight loss and its associated common path, there was an interaction between treatment group and the change in proportion of dietary saturated fat. Age was also found to influence the change in HbA1c. This model explained 13.6% of the variance of change in HbA1c.

#### Common paths

Change in weight was found to be an important factor for the change in each trait and this common path is shown in black (Fig 2). The factors identified to be important for changes in weight were treatment group, changes in physical activity and in the proportion of dietary saturated fat, with participants in the CP group having lost 3.53 kg more following the intervention than those in the SC group, an additional 0.18 g weight loss for each additional 1000 steps, and a reduction of 0.20 kg for every 1% reduction in the proportion of saturated fat. These factors, accounted for 11.9% of the variance of change in weight. Treatment group was an important factor in the change in proportion of dietary saturated fat with participants in the CP group reducing the proportion of saturated fat in their diet by 1.13% of energy compared to SC. Country and sex were also found to influence changes in the proportion of dietary saturated fat. These factors accounted for only 5.58% of the variance in the change in proportion of dietary saturated fat. None of the factors studied were identified to be important for changes in physical activity.

### Weight loss interactions with dietary saturated fat or with treatment group on changes in traits

**Fig 3:**
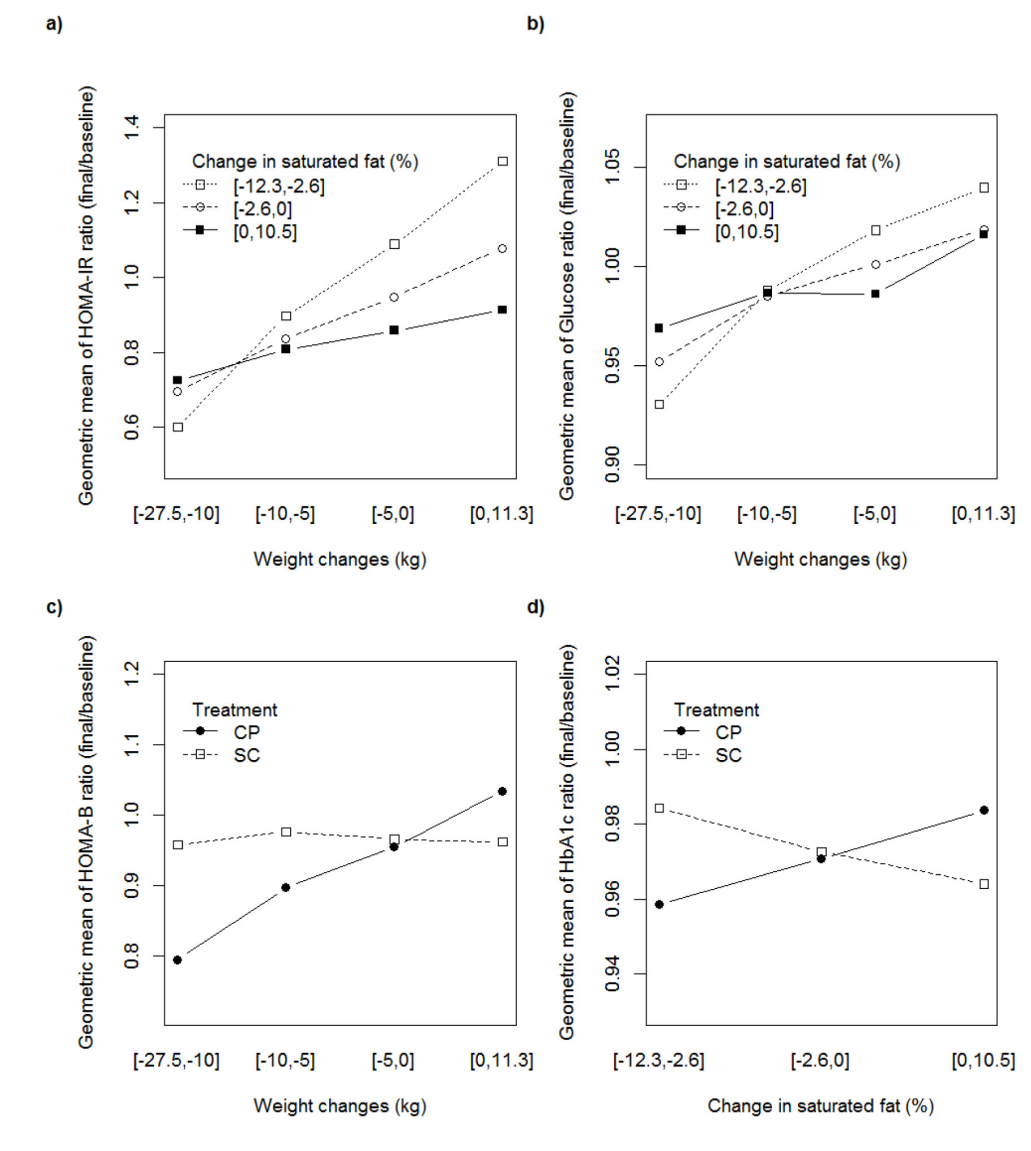
Interactions between weight loss, dietary saturated fat and treatment group on changes in traits. Plots of interactions identified in the models represented in Fig 2 are shown for **a)** the interaction of changes in weight and saturated fat on the changes in HOMA-IR **b)** the interaction of changes in weight and saturated fat on the changes in glucose **c)** the interaction of treatment group and weight changes on the change in HOMA-B **d)** the interaction of treatment group and change in saturated fat on changes in HbA1c. Each outcome measure was logged for analysis, therefore the change is represented as the geometric mean of the outcome ratio (final/baseline) where a result >1 indicates the variable was higher at the 12 month measure than the baseline measure, conversely a result <1 indicates the variable was lower at the 12 month measure than at baseline. Change in outcome is indicated on the y-axis (**a-d**) and the scale of the y-axis was chosen to represent a wide range of the outcome values between the lower and the upper quintiles. Changes in weight are shown along the x-axis (**a-c**). Changes in the proportion of dietary saturated fat are represented by the 3 curves which approximate to tertile levels in **a & b and by the x-axis in d**. The two treatment arms are represented by separate curves in **c & d**.

Interactions between weight loss and treatment group or change in proportion of saturated fat which were important factors identified by the models (Fig 2) are illustrated in Fig 3. As shown in Fig 3A, HOMA-IR was improved in most participants (represented as a HOMA-IR ratio of final/baseline <1). The greatest improvements were seen in participants who lost the most weight and achieved the greatest reduction in dietary saturated fat. There was a deterioration in HOMA-IR in those who gained weight.

#### Glucose

There was a moderate improvement in plasma glucose for most participants (represented as a plasma glucose ratio of final/baseline <1). As shown in Fig 3B, the interaction between weight change and change in the proportion of dietary saturated fat had modest effects on change in plasma glucose. For participants who increased their saturated fat intake, any weight change had little effect on plasma glucose, whereas for participants who achieved a reduction in saturated fat, weight change had more of an impact. For example, participants who decreased the proportion of saturated fat by 5%, but had no weight loss, had a slight increase in plasma glucose (1.31%), whereas those who both reduced saturated fat and lost 20 kg decreased their plasma glucose (6.23%).

#### HOMA-B

As shown in Fig 3C, there was no change in insulin secretion represented by a change in HOMA-B (final/baseline ratio = 0.98) in the SC treatment arm, for any degree of weight loss. Conversely, for participants in the CP treatment arm, HOMA-B was lower the greater the degree of weight loss. For example for participants who achieved a mean weight loss of ~5 kg, HOMA-B was decreased (final/baseline ratio = 0.91) and those that achieved a greater weight loss of ~15 kg decreased their HOMA-B further (0.78).

#### HbA1c

HbA1c (Fig 3D) was improved overall (represented as an HbA1c ratio of final/baseline <1) but greater improvements were seen for those in the CP group who reduced their saturated fat, whereas the converse was seen in the SC group.

## Discussion

This paper illustrates the novel application of graphical Markov modelling to interrogate pathways of the complex responses to a lifestyle intervention. Using the example of improvements in glycaemic traits we have shown that changes in weight, and the associated changes in physical activity and dietary behaviours, were important for the change in all traits. In contrast, after partitioning out the effects of diet, weight loss and physical activity, genetic predisposition to T2DM impacted only upon the change in insulin sensitivity and glucose, but not on insulin secretion. This finding was interesting given that at baseline there was no association between genetic predisposition and insulin sensitivity but there was an association with insulin secretion, as seen previously for SNPs associated with increased risk of developing T2DM [7, 23].

This novel method sheds light on how genetic predisposition and various lifestyle changes might contribute differentially to improvements in glycaemic traits over the course of a 12 month weight loss trial, rather than to provide definitive answers. We used an indicator for each risk factor; the T2DM-GPS for genetic predisposition and the change in number of steps and the change in proportion of saturated fat as an indication of changes in lifestyle factors for which there is convincing and probable evidence to lower the risk of T2DM [9]. Our models only explained up to 18% of the variation in change in traits over the 12 months, indicating many other factors contributed to the changes, including measurement error associated with both the outcomes and covariates. There are many possible contributors to the country-specific effects we observed in addition to obvious social, cultural and economic differences; measurements for glucose, insulin and HbA1c were performed separately in the 3 different countries, diet diaries were also analysed separately in each country and it is also possible that participants in each country received different advice and support as part of the intervention delivery or responded differently to the interventions. Other limitations included the use of proxy measures of insulin resistance (HOMA-IR) and insulin secretion (HOMA-B). The self-reported diet diary, like all dietary assessment methods may be subject to under-reporting and measurement error. However, as the assumptions of stable weight cannot be applied in a weight loss trial, it is difficult to adjust for under-reporting by usual methods and we have not attempted so here. Physical activity was measured by the short-term use of pedometers, previously shown to have limited use in measuring energy expenditure [33]. Our sample set was limited to participants for which there was 12 month outcome and genetic data available. There are also limitations to the use of our T2DM-GPS to define genetic predisposition as the SNPs provided incomplete coverage of the currently identified SNPs associated with T2DM, glycaemia, insulin secretion and insulin sensitivity [2–6, 8].

However in spite of these limitations, which might be expected to mask paths of association, we have shown that graphical Markov modelling can successfully identify the important mediators of change in glycaemic traits from a number of possible factors following a behavioural intervention. While previous studies have considered effects of diet and physical activity [34, 35] or genetic susceptibility as an effect modifier on the health impact of interventions [23, 36], this is the first analysis to model the relative contributions of genetic and a number of lifestyle factors on changes in traits. Using a T2DM genetic risk score of 34 SNPs the Diabetes Prevention Programme (DPP, n = 2843), reported a trend for a lower estimated insulin secretion (insulinogenic index) for a higher genetic risk score at baseline, but no interaction between genetic risk and treatment on change in insulin sensitivity or secretion indices following 12 months of intensive lifestyle modification or metformin treatment [36]. This intervention study supports our findings that genetic predisposition does not modify the positive changes in insulin secretion and insulin sensitivity in response to a behavioural intervention. In contrast, in a five year follow-up of participants in the Inter99 study (n = 3727) a T2DM genetic risk score of 46 SNPs was associated with increased fasting and post-challenge plasma glucose levels, an effect which was stronger in individuals who gained weight [25].

This analysis clearly shows that weight loss is the key factor for change in all traits. Changes in the proportion of dietary saturated fat exerted effects on the outcomes directly or through interactions with weight loss, highlighting the importance of diet quality to maximise reductions in the risk of diabetes, but physical activity was only important via the path of weight loss. We identified an interesting interaction between change in saturated fat intake and weight loss contributing to the change in insulin sensitivity. Despite the greatest improvements in insulin sensitivity being seen in participants who lost the most weight and achieved the greatest reduction in dietary saturated fat, overall changes in the proportion of dietary saturated fat had less impact the greater the weight loss, again supporting the notion that weight loss is the dominant factor. In the Finnish Diabetes Prevention Study, saturated fat reduction was associated with T2DM risk reduction in univariate analysis, although not when accounting for weight loss [37]. In the current study, for participants who gained weight, dietary composition became a more significant determinant of the change in insulin sensitivity. Unexpectedly insulin sensitivity decreased in participants who achieved the greatest reduction in saturated fat, given that proportion of dietary saturated fat has previously been inversely associated with insulin sensitivity [38, 39]. However, there was a strong inverse association between the change in proportion of dietary saturated fat and the proportion of carbohydrate eaten and this may explain the reduction in insulin sensitivity, particularly if the increased carbohydrate included free sugars or high glycaemic index (GI) foods [9, 40, 41]. We were unable to test the independent role of these specific dietary components as sufficient data was not available for these measures, as these nutrients are not routinely available in the dietary analysis programmes used in each country. Moreover we made a prior decision to focus on variables previously reported in systematic reviews to be linked to glycaemic traits.

We also identified that treatment group was an important determinant of change in insulin secretion in response to weight loss; insulin secretion was improved in the CP group according to weight loss, but was unaffected by weight loss in participants in the SC treatment group. These treatment effects may have been due to the path through changes in saturated fat (Fig 2), although there are other factors, including other dietary factors, which were not considered in this analysis and which may have contributed to this effect. Another interesting effect of treatment group was to moderate responses to changes in saturated fat such that larger improvements in HbA1c in participants with the greatest reductions in saturated fat were only seen in the CP group. This effect may have been mediated through the treatment effect on weight, although again, other factors not included in the model may contribute to this effect. Whilst our analysis did not aim to be confirmatory, we identified that weight loss and proportion of dietary saturated fat were the most important components taking into account this complex combination of contributing factors. Physical activity has been identified as a factor with convincing evidence for preventing diabetes, but when considered in combination with a number of other factors it was not found here to play a key role in lowering T2DM risk, although this may reflect the choice of indicator measure used. It is also possible that the effects of physical activity and dietary factors may be diluted due to measurement error, in contrast to associations involving weight which is measured with greater precision.

## Conclusion

This analysis demonstrates the novel use of graphical Markov modelling to interrogate complex systems to identify mechanistic paths for the effects of genetic and lifestyle factors important for the change in glycaemic traits during a 12 month weight management trial. In this study the paths through modifiable components relating to body weight, diet, and physical activity have a greater impact then genetic predisposition on change in glycaemic traits.

## Supporting Information

S1 Fig Variation of (a) glucose, (b) HbA1c, (c) insulin secretion and (d) insulin sensitivity at baseline by genetic predisposition score (T2DM-GPS). Data are the mean and standard error values of (a) glucose and (b) HbA1c (c) HOMA-B (%) and the geometric mean and 95% CI of (d) HOMA-IR for T2DM-GPS score category defined by the number of risk alleles per individual. For depiction in this figure, T2DM-GPS at the lower and upper ends for each trait were grouped due to small n.
(PDF)

S1 Table Type 2 diabetes SNPs which compose the genetic predisposition score (T2DM-GPS). (PDF)
