# Supplementary material for: Modelling the Interplay between Lifestyle Factors and Genetic Predisposition on Markers of Type 2 Diabetes Mellitus Risk
Source: PLoS One. 2015 Jul 8;10(7):e0131681. doi: 10.1371/journal.pone.0131681 (PMC4496090; doi:10.1371/journal.pone.0131681)
Supplement: S1 Fig — Data are the mean and standard error values of (a) glucose and (b) HbA1c (c) HOMA-B (%) and the geometric mean and 95% CI of (d) HOMA-IR for T2DM-GPS score category defined by the number of risk alleles per individual. For depiction in this figure, T2DM-GPS at the lower and upper ends for each trait were grouped due to small n. (PDF) [file pone.0131681.s001.pdf]

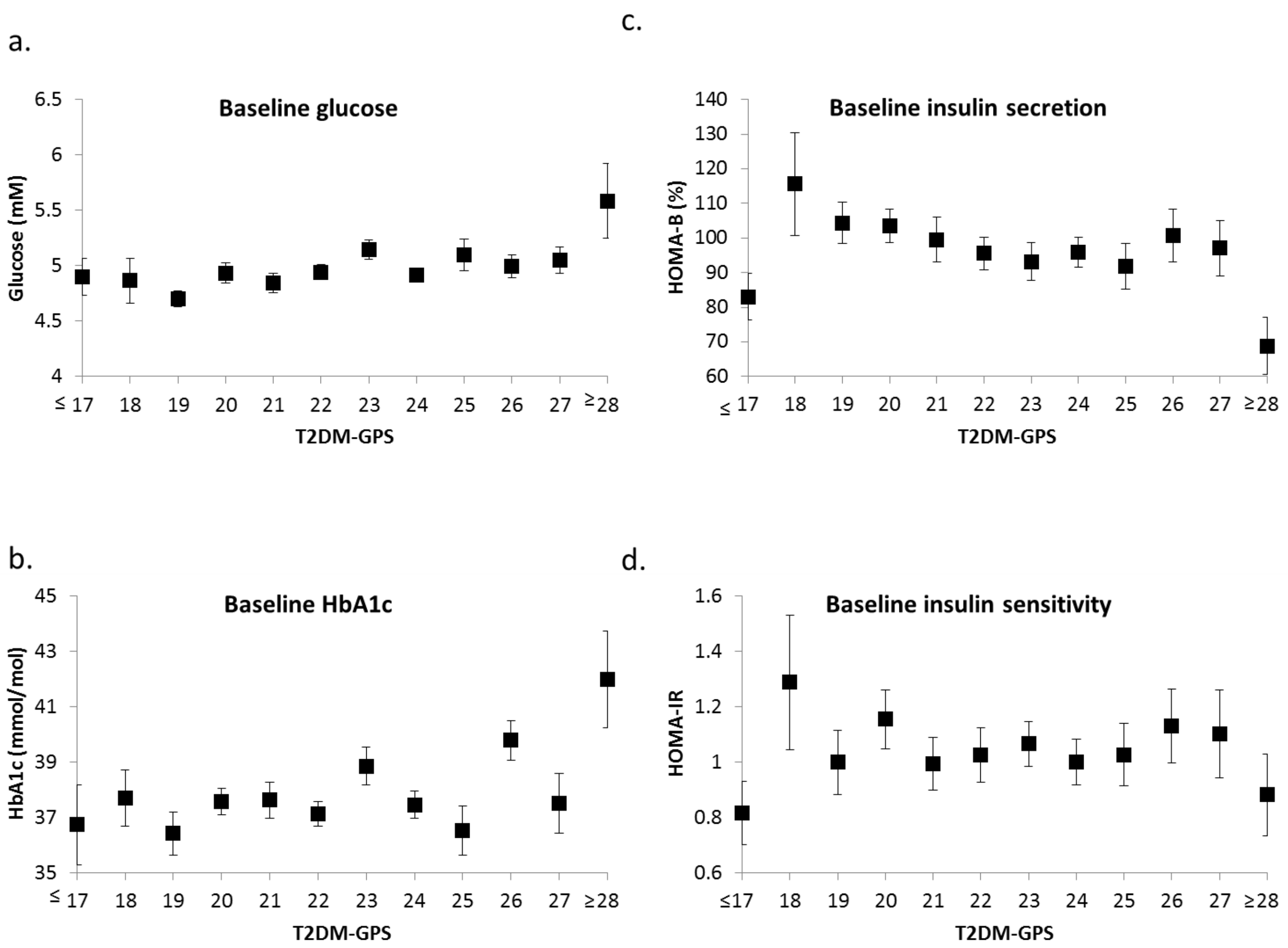

Figure S1. Variation of (a) glucose, (b) HbA1c, (c) insulin secretion and (d) insulin sensitivity at baseline by genetic predisposition score (T2DM-GPS).
