# Supplementary material for: Modelling the Interplay between Lifestyle Factors and Genetic Predisposition on Markers of Type 2 Diabetes Mellitus Risk
Source: PLoS One. 2015 Jul 8;10(7):e0131681. doi: 10.1371/journal.pone.0131681 (PMC4496090; doi:10.1371/journal.pone.0131681)
Supplement: S1 Table — (PDF) [file pone.0131681.s002.pdf]

Table S1. **Type 2 diabetes SNPs which compose the genetic predisposition score (T2DM-GPS)**

SNP	Gene	Location	Risk allele	Risk allele F	SNP (LD R^2)*	Glycaemic trait†
**rs4616635**	ADAMTS9	Intergenic	C	0.75	rs4607103 (0.91)	
**rs13028240**	BCL11A	Intergenic	G	0.69	rs10490072 (0.96)	
**rs12779790**	CDC123, CAMK1D	Intergenic	G	0.18		
**rs7756992**	CDKAL1	Intronic	G	0.26		FPG (817)
**rs10811661**	CDKN2B	Non-protein coding	T	0.85		FPG (817) FPG-A (787)
**rs564398**	CDKN2B	Non-protein coding	T	0.68		FPG (817)
**rs1153188**	DCD	Intergenic	A	0.73		
**rs10838240**	EXT2	Intronic	T	0.29	rs3740878 (0.95)	
**rs9939609**	FTO	Intronic	A	0.40		FI-U (817)
**rs563694**	G6PC2, ABCB11	Intergenic	T	0.66		FI-U (787) FPG-A (787) HOMA-B (558) FPG (558), HbA1c (924)
**rs4607517**	GCK	Intergenic	A	0.16		2hGlu (817) FPG-A (787), Hba1c (924)
**rs7501939**	HNF1B (alias TCF2)	Intronic	T	0.38	rs757210 (0.81)	
**rs2074314**	KCNJ11	Intergenic	G	0.41	rs5215 (0.95)	
**rs1801282**	PPARG	Missense (pro/ala)	C	0.85		FI-A (817) FPG-A (787)
**rs13266634**	SLC30A8	Missense (arg/trp)	C	0.68		FPG-A (787) FPG (558)
**rs17036101**	SYN2, PPARG	Intergenic	G	0.93		FI-A (787)
**rs4132670**	TCF7L2	Intronic	T	0.27	rs7903146 (0.91)	FI-U (817) FPG-A (787) FPG-A (787) HOMA-B (558)

**rs7578597**	THADA	Missense (Thr/Ala)	T	0.90		
**rs1353362**	TSPAN8, LGR8	Intergenic	C	0.24	rs7961581 (0.96)	
**rs9472138**	VEGFA	Intergenic	T	0.28		
**rs780094**	GCKR	Intronic	C	0.39		FPG-A (787) FP-A (787)

* SNP reported in previous studies {Zeggini, 2007 #347;Zeggini, 2008 #342;Voight, 2010 #587;Sladek, 2007 #350;Saxena, 2007 #603;Prokopenko, 2009 #341;Dupuis, 2009 #558}

† Locus has been associated with the glycaemic traits in previous studies {Scott, 2012 #817;Manning, 2012 #787;Dupuis, 2009 #558;An, 2014 #924}

FI-A: Fasting insulin adjusted for BMI; FI-U: Fasting insulin unadjusted for BMI; FPG: Fasting plasma glucose; 2hGlu: Plasma glucose 2 hours after an oral glucose tolerance test; FPG-A: fasting plasma glucose adjusted for BMI
